# Rehabilitation, the disciplining of the body, and disability identity: Reflections from psychotherapy with disabled people

**DOI:** 10.4102/ajod.v13i0.1505

**Published:** 2024-11-08

**Authors:** Brian P. Watermeyer

**Affiliations:** 1 Department of Global Health, Faculty of Medicine and Health Science, Stellenbosch University, Cape Town, South Africa

**Keywords:** disability, rehabilitation, psychoanalysis, psychotherapy, mental health, critical rehabilitation studies, disability empowerment, disability identity

## Abstract

**Background:**

Recently, attention has been paid to how rehabilitation not only provides medical treatment and instrumental skills but also impacts psychological well-being and identity. We all have psychological structures that discipline the self, enforcing norms internalised during early life and exacting judgments when we fail to ‘make the grade’. In cases of congenital disabilities, rehabilitation interventions may span many years, involving strict programmes of therapy, exercise and self-discipline. These regimes may align with internalised rules in harmful ways, as striving for functional improvements takes on a moral dimension, affecting psychological health and empowered disability identities.

**Objectives:**

This study explores rehabilitation by examining the experiences of adults with congenital disabilities, who have undergone childhood medical and rehabilitative interventions.

**Method:**

This study was based on the experience of a psychoanalytic psychotherapist working with adults with disability, and presents composite case material to illustrate how interactions with medical authority figures, such as rehabilitation professionals, can have a formative influence on self-identity and entitlement to inclusion.

**Results:**

The findings vividly reflected how ‘medical socialisation’ created meanings of disability that were enacted and repeated well into adulthood.

**Conclusion:**

The discipline of rehabilitation still has much to do in examining its value-laden assumptions and practices, and how these may shape the internal and relational worlds of people with disability.

**Contribution:**

This article contributes to the debate in critical rehabilitation studies, focussing on the issue of constructions of disability which may be communicated to people with disability, with implications for self-advocacy and the growth of the disability movement.

## Introduction

Like other biomedical interventions, rehabilitation has traditionally viewed itself as a discipline that unambiguously benefits people with disability, in a way which is not complicated by ideology or vested interest (Botha & Watermeyer 2022). More recent accounts have begun to query this position, opening new critiques of how rehabilitation practice has implications for how disability is constructed across society, and the implications of this for social change (Gibson 2016). This in turn resonates with aspects of critique from the disability movement and the discipline of disability studies, which can be traced back to the historic protest at the congress of Rehabilitation International (RI) in Winnipeg in 1981 (Swain et al. 1993). There, delegates with disability demanded strong benchmarking of disability representation at the event, with the denial of this demand leading to the formation of Disabled People International (DPI) a few years later. Over the past decade, the emergence of the sub-discipline of Critical Rehabilitation Studies (CRS) has added to the growing critical discussion (Gibson 2016; Sullivan 2005).

As one assesses the layered reality of disability-related disadvantage, levels of analysis vary from ideological and material to relational and intra-psychic. Unfortunately, however, research in these different domains can appear splintered, missing an opportunity to explore how the various strands of socially engendered disadvantage interact in the context of real lives (Watermeyer 2013). With the critique of rehabilitation in mind, this article presents an account of the life of a man living with a physical disability, which contrasts the anonymising, inevitably reductive public health-oriented view of his social predicament with the felt reality of his personal, inner life. At issue is the reality of the lifelong socialisation of many people with disability in a world saturated with ableist assumptions and signals – a world in which rehabilitation practices and professionals sometimes play an influential role.

The motivation for this article stemmed from my experience as a psychoanalytically oriented psychotherapist working with clients with disability. This role provides an exceptional opportunity to witness the workings and implications of ableist socialisation, including that involving health interventions, while also demonstrating how the complexity of individual disability experience confounds the categories of research in sociology and public health. As will become clear, I regard depth-oriented psychotherapy as one crucial route towards empowerment of the disability community, based on its potential to unpack and debunk deeply embedded ableist assumptions about the self and the world borne of life in a discriminatory society (Watermeyer 2012). Clinical accounts from psychotherapy with people with disability have an immense amount to teach us about how ideology settles in at the level of the intra-psychic, and from there becomes enacted in relationships with the self and the world, which can make it harder to escape the clutches of negative beliefs about life with disability. To demonstrate this, and add to debates on how rehabilitation practice can shape disability identity, I present a composite case history of a psychotherapeutic process with an adult with a physical disability, combining and disguising material from a host of therapies I have been involved in, in accordance with ethical principles of client confidentiality in psychoanalytic scholarship (Gabbard 2000). While only one case study is presented, it is one that carries elements which appear frequently in my work, and I believe it may provoke fruitful discussion on these issues. I begin, though, by describing my own positionality as a disability studies scholar and a person with disability – identities that both require reflexive consideration as I approach this material. Thereafter, I move into a discussion of the case material, considering its implications for rehabilitation theory and practice.

## Introducing my positionality

I am trained as a clinical psychologist, and employed primarily as a disability studies academic, while working part-time as a psychoanalytically oriented psychotherapist working with people with disability in private practice. I also live with a disability myself, in particular, severe visual impairment. While my background in psychology and psychoanalysis means that I approach the question of disability inequality from a psychological point of view, this often contrasts with my academic environment, which is a department of global health, within a health sciences faculty.

I offer these details because of how my circumstances provide me with varying, although perhaps complementary, perspectives on disability-related social disadvantage, as well as the psycho-emotional aspects of living with impairment. In my ‘day job’ at university I am steeped in what might be termed a public health view of disability, which is to a large extent dominated by quantitative data on the social circumstances of people with disability, in crucial areas such as access to healthcare, employment, transportation and other essential resources and services. These data are essential to our understanding of the systemic nature of disadvantage, rendering imperatives for change at the level of policy and service design (WHO 2011, 2022). Yet, such knowledge also provides a view of the life-worlds of people with disability which is, to some extent, anonymising. By necessity, the need to identify categorical distinctions means creating divides between the ‘haves’ and ‘have nots’ that smooth over the vast complexity in experience which reflects the reality of life with disability in an ableist society. While the deprivation of essential resources is a contravention of basic human rights, which demands redress at the highest level, as a categorical descriptor it homogenises the experiences of all, flattening the radical diversity of lives with disability.

Again, this is utterly necessary, but shows only an aggregated and depersonalised level of reality, while eliding others. For example, what this form of knowledge can do is to propagate the idea that access to, say, reasonably appropriate healthcare somehow makes the problems, the quandaries, of the societal experience of disability ‘go away’; this is seldom the case, and reflects a view which is, at best, incurious. Such an individualising perspective is still discernible in the WHO’s 2030 action plan for developing rehabilitation services (WHO 2017). By its nature, disability confounds biomedicine’s understandable need for reductive sense-making, scrambling its narrow ideas on where ‘the problem’ of the social adversity experienced by people with disability lies.

In stark contrast to the picture aforementioned is the view I have of disability inequality from my therapist’s chair in my home consulting room. Here, I literally see the contextual experience of disability from the opposite angle – from that of individuals often undergoing lifelong experiences of discriminatory treatment not only at structural and systemic levels but also at the level of the personal and relational. Furthermore, the issue at hand involves not only the material and relational realities of what occurs ‘out there’ in the community but also the psychological impressions these realities leave on the inner world, which are often long-lasting (Watermeyer 2013). Here, inner emotional experience and development are interwoven over decades with ableist experiences in families, institutions and the community, in a manner that can at times make the public health perspective seem like an absurd simplification. For example, in the life of an adult with a congenital impairment such as cerebral palsy, the developmental milieu from earliest infancy has been, to a greater or lesser degree, shaped by ideas and emotions surrounding disability prevailing within the family (Parens 2006, 2009). Our shared legacy of disability segregation means that very few people have had the opportunity to live alongside persons with a range of impairments, who may be living fulfilling, flourishing lives. Consequently, parents faced with the unexpected reality of a newborn with cerebral palsy will respond, both consciously and unconsciously, in a variety of ways. The lack of essential early childhood intervention support for such parents can lead to the enactment of commonly held ambivalences and prejudices about the life potential of individuals with disability, starting from the earliest period of infancy (Harris & Wideman 1988; Lussier 1980; Watermeyer 2002). But even when support is available, as noted earlier, the ‘problems’ of disability are not ‘solved’. Sustained, depth-oriented psychotherapy provides a window into the internal worlds shaped by such circumstances, and how identity itself is all too often entangled with ableist beliefs. The process of uncovering these realities is by necessity slow, as many memories are sealed closed by associated trauma, which must be processed in order for internal shifts to take place. Of course, this work is also expensive and, lamentably, the prioritisation of mental healthcare to the disability community remains woeful, reflecting a lack of insight into the gravity of the exact issues I describe here. The notion of ‘disability empowerment’ is at times used loosely and has varying definitions (Marks 2002). A psychoanalytic view emphasises that the growth of personal power rests on solid, internal foundations, borne of a debunking of internalised ableist ideas, as well as the processing of the possibly traumatic experiences with which they are associated (Marks 2002; Watermeyer & Swartz 2016).

## Medical socialisation

A further possible aspect of growing up with a congenital disability pertains to what we may describe as ‘medical socialisation’; in other words, the ways in which acute or sustained exposure to medical institutions and professionals may have a formative influence on the lives of people with disability (Tremain 2017). Medical and rehabilitation professionals often appear in the lives of people with disability at times of acute trauma, such as after the onset of an acquired disability, or after the birth of an infant with a congenital impairment (Sullivan 2005). These are moments, or periods, during which anxiety is high, and the future uncertain. As discussed, parents in such circumstances have probably not had the experience of adults with fulfilling lives who live with the same impairment as their child, as is also the case with an adult with an acquired disability such as paralysis. Consequently, these individuals may feel deep uncertainty about what sort of future is possible for their child or themselves – questions to do with ‘where I belong now’, ‘what I can hope for’ and ‘what disability does to my identity’. In this scenario, the overt and non-verbal signals received from health professionals about the implications of disability for a fulfilling life can be very powerful indeed (Parens 2009). For children growing up with congenital disabilities requiring ongoing rehabilitation regimes, a relationship with a rehabilitation therapist may have a formative influence on the child’s incipient disability identity, with potentially far-reaching implications for self-worth and emotional well-being in adult life.

## Critiquing rehabilitation

Rehabilitation has, like other health disciplines, historically been regarded as value-neutral (Marks 1999). In other words, the work of rehabilitative therapy has been understood as unidimensionally positive – the ‘good work’ of ameliorating the functional limitations of impairment. This view, when set against several decades of critical disability studies scholarship, proved to be a naïve one (Gibson 2016). The ideological terrain surrounding disability is always fraught with tensions to do with control and normalisation, the perennially challenging question of human difference and dilemmas of identity (Davis 2001). Wandering into this melee, rehabilitation cannot help but assume a host of ideological positions, often not explicit, but hidden in the unarticulated underpinnings of practice (Ravaud & Stiker 2001). The recent emergence of the field of CRS has signalled the beginning of a drive to develop and consolidate critiques of rehabilitation, which have been with us for some years (Botha & Watermeyer 2024). The simple truth that rehabilitation science’s rasion d’etre is to correct, to put right, to repair, is both fundamental and inescapable (Ravaud & Stiker 2001). Consequently, it is not something for which the discipline needs to apologise, but it does present a responsibility to examine its ideological foundations and implications. In short, the way in which rehabilitation is accomplished will inevitably be entangled with how disability is constructed in society, and, crucially, how people with disability feel about themselves and the world.

## Case material

Having set the scene, I now move to a description of a composite client from my psychotherapy practice, holding together the stories of several clients whose early lives have been shaped by rehabilitation. In terms of the ethical responsibility to protect client confidentiality, I employ the composite method, while also fully subscribing to the International Psychoanalytic Association’s principles for scholarship based on psychoanalytic work (For more details on anonymisation to protect client confidentiality, see: https://files.taylorandfrancis.com/ripa-patient-anonymization-author-checklist.pdf.).

### Rafik’s story

Rafik is a 48-year-old Muslim man living with cerebral palsy, who has been in weekly psychotherapy for 4 years. He is mobility impaired, and mostly uses a wheelchair, although is also able to walk short distances with crutches. He works as a financial officer in a small retail company, an occupation with few prospects, around which he remains ambivalent.

### Growing up

Rafik’s childhood and young adulthood reflected ongoing, often strict and demanding rehabilitation regimes, especially physiotherapy. His earliest memories are of being encouraged to try harder to walk, as his parents and physiotherapist presented what he termed a ‘united front’, in their preoccupation with normalising his mobility as much as possible. Therapy sessions occurred three times weekly for extended periods, and he recalls levels of exertion and pain which drained him of both physical and emotional energy. From these earliest experiences, however, the ‘work’ of therapy took on a value-laden aspect, in which he came to experience it as a duty he owed, in particular, to his parents. Both his mother and father spoke regularly of their hope that he would reach his ambitious therapeutic goals, and how happy this would make them. From here, he noted ‘it was easy to feel that there was something, like, really wrong with me that I needed to put right’. The imperative to try harder, to ‘be better’ also interacted with the family’s religious values, adding to the sense of a moral duty to heal, and the germ of an idea that even to Allah his essential nature was somehow unacceptable.

Like many other families in such circumstances, Rafik’s parents and siblings sincerely wanted the best for him, and their encouragement was motivated by love (Ferguson 2001; Harris & Wideman 1988; Parens 2009). Yet, it is also inescapable that we are all socialised, to some extent, into a belief that life with disability, as well as disability itself, is somehow wrong, undesirable or spoiled. This family was no different, and these unarticulated beliefs were everywhere in Rafik’s young life, mixing love with something which, at a deep level, came to feel like some form of rejection. The expressions of love, manifesting in an imperative that Rafik be ‘repaired’, could not but carry with them a foundational struggle with, even abhorrence of, difference. This is a terrible conundrum for all and underscores the gravity and stubbornness of internalised oppression as an issue in the empowerment and political mobilisation of people with disability (Thomas 2001). Unless carefully explored and discussed, the intentions of impairment-based physical rehabilitation are simply antithetical to the acceptance of human difference; it is the difference that we fight, day in and day out, in the therapy room, and it must therefore clearly not be our friend. Echoing accounts from auto-ethnographic disability studies literature on rehabilitation, Rafik described how it felt like ‘I had to try as hard as I could to not be like I was’ (French 1993). Obviously, this relational predicament flies in the face of the intentions of most parents, who seek to create an environment of acceptance for their child, which lays the groundwork for self-acceptance and self-worth in later life.

Besides the religious values which the imperative to walk intersected with, Rafik’s family also manifested a common culture of stoicism, in which expressions of vulnerability were frowned upon. His school experiences, which involved a heavy valuing of sporting prowess, added to these signals, causing him to believe that he would be rejected or shamed if he expressed negative emotions such as sadness or distress. The internal reality though, was that from an early age his life regularly carried profoundly distressing experiences.

He faced othering and bullying at school, felt afraid and uncertain about his future, and regularly suffered from physical pain, exhaustion and loneliness. It is likely that the culture of ‘bearing up’ and coping in silence existed within this family before it was visited upon by disability, but the presence of Rafik’s physical impairment seemed to render the tendency more acute. The imperative to not show distress often relates to anxiety within the observer about being confronted with the pain of another, with such expressions consequently being ignored or shamed. We all carry an unconscious store of our own traumatic feelings and memories, accumulated over lifetimes of developmental challenges, losses and traumas (Frosh 1991). Our need to maintain distance from these formidable aspects of our internal worlds renders an aversion to expressions of distress in others, as these may awaken our own vulnerabilities, threatening our emotional security. But in the case of disability, the stakes of this dynamic are raised, for two reasons. Firstly, it is argued that people with disability often serve as containers for the unwanted, shameful projections of the non-disabled majority, positioning this group as an unwanted other (Marks 1999; Watermeyer 2013). Secondly, there is a reality to the proposition that people with disability do, in fact, often experience extreme suffering, by dint of the systemic exclusion, stigma and discrimination which is pervasive in so many societies, not to mention the very real issues of functional limitation, fatigue and physical pain. For both of these reasons, as a society, we really do not want to know how people with disability feel, as it may overwhelm us, and also evoke feelings of guilt because of our complicity in maintaining a social order which is shot through with harm and injustice.

In Rafik’s life, the foregoing combined, enforcing a far-reaching silence on his emotional life, both from others and ultimately even from himself. As a child growing up with a severe impairment situated in an ableist and excluding world, he needed specific, substantial and ongoing emotional support, almost certainly more than the average child. Yet, with painful but familiar irony, my understanding is that his disability led to the opposite extreme, where anxiety within his parents about his difference, dressed up as a loving imperative to work harder at rehabilitation, left him internally abandoned in a life of daily emotional trauma. Let me reiterate that these parents, like most parents, had only the best intentions for their child, as they faced the physically and emotionally challenging reality of raising a child with a significant physical disability (Ferguson 2001). Sustained mental health support, based on early childhood intervention to assist them through the early years of Rafik’s life, as well as to process their own unacknowledged feelings about disability, might have made a world of difference. However, as noted earlier, mental healthcare for people with disability and their families remains poorly understood and inadequately prioritised.

### Adulthood

The man I met in his early forties still clearly carried the struggles and unresolved conflicts of his early life. While he had, despite discrimination and other forms of adversity, managed to achieve a post-school qualification in accounting and find relatively secure employment, he was brought to my consulting room by ongoing struggles with depression, harsh self-judgement and deep feelings of inferiority. His assessment of himself described a litany of failures, including in his career, his personal relationships and love life, and as a son and sibling within his family. Rafik’s story was one of a lifelong struggle for self-acceptance.

At the heart of these feelings was a conviction that, in some fundamental way, ‘I am wrong the way I am, and must be better’. As our conversations grew, links between these feelings and what had occurred over his childhood within his family and his experience of rehabilitation became increasingly evident. What the implicit (although inadvertent) messaging from his parents and therapists had left him with was a foundational, unconsciously held belief that he was to blame for his disability, and for the anxiety and distress that it had brought to his family. This logic is not hard to understand; the imperative to ‘put it right’ through work in rehabilitation settles as an idea that, if it is my duty to solve the problem, then the ongoing prevalence of that problem must be my fault, my failing. Needless to say, these feelings about one’s inherent damage, and a sense of having failed to repair it, form a very unsteady foundation for moving out into the world, especially a world that demands eloquent self-advocacy from people with disability if exclusion is to be challenged. At base, the capacity for such healthy entitlement to inclusion relies on an authentically felt belief that it is ‘OK’ to live with disability; that it is ‘OK’ to be the way I am (Watermeyer & Swartz 2008). In Rafik’s life, as is the case with people with disability everywhere, much in his socialisation signalled exactly the opposite, and rehabilitation services were clearly implicated in this. Rehabilitation’s drive to ‘put him right’ had been delivered and internalised with no balancing validation of difference, leaving Rafik in a quandary in which he experienced his ‘true’ self as damaged and unacceptable to the world. Although walking was painful and exhausting, it was the only choice bringing the possibility of approval, creating a widening internal split between his authentic experience and the person he felt he must strive to be.

The regime of silence around vulnerability that characterised Rafik’s family life had, in adulthood, taken up residence in his internal world. What this meant was that experiences of struggle, sometimes relating to ableist treatment and the effects of impairment, were met in his internal dialogue with punitive self-blame, shaming and invalidating his daily difficulties. I have written elsewhere about how growing up in a discriminatory world can lead to people with disability developing harsh super-egos – the super-ego is Freud’s term for our inner critic, or moral conscience (Watermeyer 2013). Quite simply, harsh judgements ‘out there’ translate into similar judgements ‘in here’, because it is only through experiences of acceptance by others that we can grow in self-acceptance (Winnicott 1965), and often the opposite case pertains. What this means is that many people with disability, like Rafik, live in a state of fighting a battle on two fronts, against the stigma, criticism and othering of the social world, as well as the voices of internal judgement.

Returning to the notion of empowerment, I noted earlier that personal power is built on the processing of emotional trauma. In short, developing the self-compassion needed in order to express healthy entitlement requires a thorough examination and integration of the ways in which we have been hurt. People with disability who, like Rafik, have been drawn into a dynamic of self-blame through their socialisation are, until receiving appropriate support, unable to fully recognise what they have suffered at the hands of a world consistently ambivalent about disability. Put another way, the nature of ableist socialisation makes it difficult for people with disability to grieve for what was lost through lives of socially engendered exclusion, a state which has been termed melancholic suspension (Cheng 2000). Preclusion from grieving means being unable to access the just anger which can flow from it, leading to political mobilisation, personal power and social change (Watermeyer 2017; Watermeyer & McKinney 2022). The work of emotional exploration such as that performed with Rafik was aimed at precisely this form of emancipation and subversion.

## Conclusion

### Rehabilitation culture

As discussed, over recent years a turn has become discernible in rehabilitation scholarship and practice towards greater reflexivity and a recognition that therapeutic interventions are always embedded in ideology. Part of this has involved a greater focus on the meaning of therapeutic relationships, and how powerful these can be in shaping identity. In addition, the revolutionary swing towards community-based rehabilitation, with its centralising of community integration and empowerment, has gone a long way to addressing the individual, medicalising logic at the heart of the original rehabilitation project. However, the difficulty of addressing rehabilitation’s internal contradictions should not be underestimated. Rehabilitation science, like biomedicine itself, cannot avoid separating the ‘damaged’ from the ‘whole’, the ‘vulnerable’ from the ‘strong’, the ‘fortunate’ from the ‘unfortunate’. All of this militates against what we might term human meeting – that is, the compassion borne of an authentic recognition of the universality of human vulnerability, which is the essential basis for truly integrative change in the disability arena (Davis 2002).

Rehabilitation professionals are in an excellent position to model acceptance and validation of human differences, but achieving this will demand concerted and nuanced change to both philosophy and practice. An essential point of departure is training that offers a deeper emphasis on relational aspects of practice, addressing not only instrumental skills but also the personal and emotional relationships which young practitioners have with disability as an axis of human difference. Packed training programmes and under-resourced service installations contribute to a reductive and functionalist view of rehabilitation goals, allowing un-thought enactments of ableist values in clinical practice. The only route to a deeper understanding of the emotional currents and ramifications implicit in rehabilitation relationships requires that practitioners be provided with opportunities for supported introspection, in which their own feelings, motivations and needs surrounding their disability work may be brought to light (Watermeyer in press). Such insight allows for relational skills sufficiently nuanced to offer interventions which aim to correct ‘flaws’, while also continually affirming personhood, choice and a self-identity that can be whole no matter one’s divergences from biomedical normalcy. Practitioners need to understand, first and foremost, their entanglement in a world of ideological propositions about disability, which have deep and lasting implications for the self-identity of people with disability, while recognising the gravity of their role. Rafik felt that his humanity had somehow become lost in the functional regimes of health professionals; relationships that begin by taking seriously the reality of our shared humanity are more likely to grow a secure, integrated sense of self in all of us, clients and practitioners alike.
